# Diversity of rice rhizosphere microorganisms under different fertilization modes of slow-release fertilizer

**DOI:** 10.1038/s41598-022-06155-1

**Published:** 2022-02-17

**Authors:** Yulin Chen, Panfeng Tu, Yibin Yang, Xinhai Xue, Zihui Feng, Chenxin Dan, Fengxian Cheng, Yifan Yang, Lansheng Deng

**Affiliations:** 1 Department of Plant Nutrition, College of Natural Resources and Environment, South China Agricultural University, Guangzhou, 510642 People’s Republic of China; 2 Department of Horticulture, Zhongkai University of Agriculture and Engineering, Guangzhou, 510225 People’s Republic of China; 3 Dongguan Yixiang Liquid Fertilizer Co., Ltd, Dongguan, 523125 People’s Republic of China

**Keywords:** Biodiversity, Bacteria, Agroecology, Biodiversity

## Abstract

The application of slow-release fertilizer is an effective way to satisfy the demand for nutrients of crops. The objective of present study was to investigate the microbial community characteristics in rice rhizosphere soil under different fertilization modes of slow-release fertilizer. Three fertilization modes of slow-release fertilizer, i.e., (CK) manually broadcasted on the soil surface at 300 kg·ha^−1^ before transplanting and then same fertilizer rate was applied at the same way one week after transplanting; (SF) 10 cm depth mechanized placement at 600 kg·ha^−1^ during the transplanting; (DSF) 10 cm depth mechanized placement at 480 kg·ha^−1^ during the transplanting, were adopt in the field experiment. The results showed that SF and DSF treatments promoted richness (ACE and Chao1 values) and diversity (Shannon value) of rice rhizosphere microorganisms compared with CK treatment. Compared with CK, SF treatment increased relative abundances of Planctomycetes and decreased relative abundance of Nitrospirae, DSF treatments increased relative abundances of Deltaproteobacteria. Moreover, higher relative abundances of *Paenibacillus* and *Sphingomonas* were recorded in DSF treatment than CK. In addition, the partial factor productivity (PFP) deep placement of slow-release fertilizer treatment was significantly higher than that of CK treatment. DSF treatment increased the yield by 16.61% compared with CK treatment while reducing fertilizer input by 20%. In conclusion, compared with broadcasting, deep placement of slow-release fertilizer could improve the structure, distribution, and diversity of the microbial community in rice rhizosphere soil, and increase the utilization rate of fertilizers, and increase rice yield.

## Introduction

As the staple food of more than half of the world’s population, rice is mainly grown in India, China, and other countries. China is the second-largest rice-growing country, but its rice output is the largest in the world^1^. However, with the rapid growth of the world’s population, rice has become increasingly important in ensuring food security^2^. So far, chemical fertilizers are still one of the main means to increase rice yield^3^. In China’s conventional rice planting process, rice growers do not apply fertilizer until the rice transplantation is completed^4^. To obtain higher rice yields, rice growers will broadcast excessive fertilizer^5^. However, broadcast fertilizer is not a good way of fertilization. Two-thirds of the fertilizer cannot be absorbed and used by rice in wetland rice production^6^. The wasted fertilizer increases the input cost, thereby reducing the profit and net income of rice growers, but also causes harm to the surrounding environment. At present, most of China’s wetland rice has begun to be planted by machinery. The most popular one is the machine that synchronizes sowing and fertilization, which saves the time and cost of manual fertilizer application, thereby increasing the profit and net income of rice growers^7^. Broadcast fertilizer is the least effective of many fertilization methods. Fertilizer broadcast increases the contact area between fertilizer and soil on the soil surface, a large amount of fertilizer is fixed by the soil, and the dispersed fertilizer is not conducive to full utilization by rice^8^. However, the machinery can concentrate and deep apply fertilizer on the side of the rice, which greatly reduces the contact area between the soil and the fertilizer, avoids the loss of nutrients caused by runoff, and the rice can also make full use of the fertilizer^9^. Therefore, the mechanical side deep fertilization is a better choice for the application of wetland rice planting.

In China’s conventional rice fertilization process, ordinary chemical fertilizers have a short fertilizer effect time, and it is necessary to broadcast 3–4 times of fertilizer^10^. This method of fertilization not only requires a lot of labor but may also cause a series of environmental problems due to excessive fertilizer input, such as heavy metal pollution caused by soil acidification and groundwater pollution^11–13^. The emergence of slow and controlled release fertilizers provides a new way for more environmentally friendly and effective rice fertilization. Nowadays, there are more and more varieties of slow and controlled release fertilizers. There are single-element slow and controlled release fertilizers and multi-element slow and controlled release compound fertilizers^14^. Trenkel et al.^15^ put forward three conditions that should be met as a slow and controlled release fertilizer, that is, the release amount shall not exceed 15% in 24 h in water at 25 °C; the release amount shall not exceed 75% in 28 days; at least 75% release within the set fertilizer effectiveness period. This standard is the current internationally recognized standard for coated slow-release fertilizers^16^. Slow and controlled release fertilizers are divided into slow-release fertilizers and controlled-release fertilizers, but there is no clear official distinction between them. It is generally believed that slow-release fertilizers include controlled-release fertilizers, and fertilizers with physical barriers are classified as controlled-release fertilizers^15^. Slow and controlled release fertilizers are generally divided into condensation products of urea aldehyde, fertilizers with physical barriers (coated or incorporated into the matrix), and super granules^16^. The study of Tang et al.^17^ showed that when fertilizers were broadcast all at one-time, the soil available nitrogen content of urea treatment was always lower than that of slow and controlled release fertilizer treatment in the first month after fertilization. A three-year experiment in Northeast China also showed that the rice yield of the treatment with slow and controlled release fertilizer was always higher than that of the treatment with urea^18^. In addition, the nutrient release rate of slow and controlled release fertilizers is affected by the placement position. Regardless of the type of slow and controlled release fertilizer, the nutrient release rate of the slow and controlled release fertilizer applied on the surface is faster, and the nutrient release rate of the deeply applied slow and controlled release fertilizer is more in line with expectations^19^. Therefore, it is good to apply slow and controlled release fertilizer deeply for only applying fertilizer once during the entire growth period.

The rhizosphere is a specific habitat for microorganisms in the soil ecosystem^20^. As an important component of the soil environment, soil microorganisms have an inseparable relationship with the growth and development of plants^21^. The beneficial microorganisms in the rhizosphere soil of rice can provide more nutrients for rice by fixing nitrogen and dissolving phosphate and can also resist diseases^22^. Thus, ensuring the diversity of rice rhizosphere soil microorganisms has an important impact on rice’s normal growth and development. However, the activities of soil bacterial communities are often affected by many factors, such as the type of fertilizer, the mode of fertilization, the level and frequency of fertilization^23,24^. The study of Masahito Hayatsu^25^ pointed out that slow and controlled-release nitrogen fertilizers can improve the rhizosphere soil microbial community. The study showed that compared with no-tillage, traditional farming would affect the bacterial community structure of rice^26^. Mechanical deep fertilization has less damage to the soil, which is between no-tillage and traditional farming. However, the current mechanical deep fertilization mainly studies rice yield, economic benefits, nitrogen utilization, and loss, and there is no relevant report on the impact of deep mechanical fertilization on the bacterial community structure of rice rhizosphere soil^4,27,28^. The present study was conducted in Guangdong (a major rice-producing province in South China) to examine the effects of deep fertilizer placement on bacterial community structure diversity in rice rhizosphere soil, grain yield, and the partial factor productivity of applied fertilizer (PFP) to provide a basis for scientific fertilization and irrigation, improving farmland fertility and maintaining soil microbial diversity, etc.

## Results

### Evaluation results of the high-throughput library of soil bacterial communities

**Figure 1:**
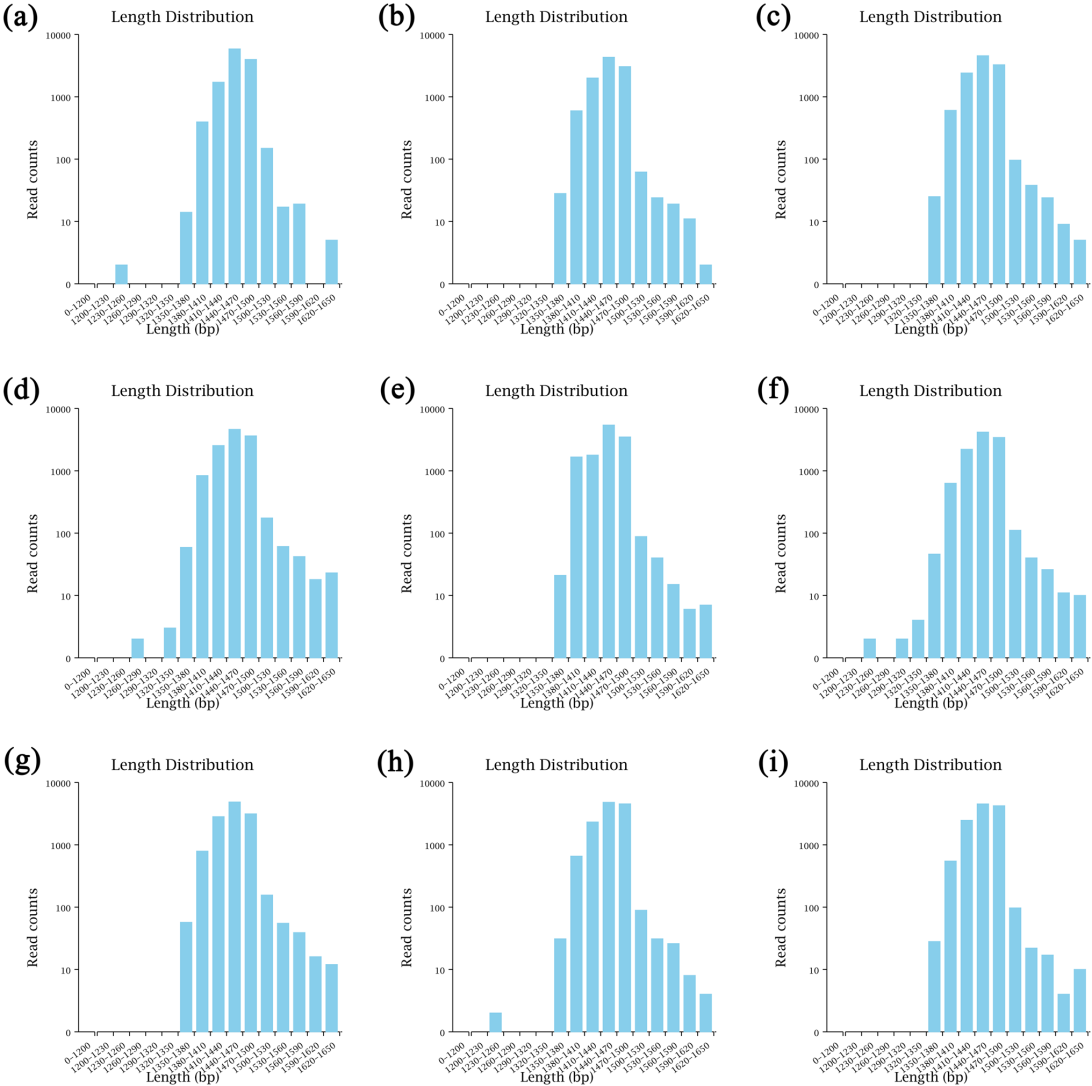
The number of sequences within the corresponding length range in each sample after quality control filtration. (**a**–**c**) for CK; (**d**–**f**) for SF; (**g**–**i**) for DSF.

Based on bacterial 16S rRNA sequence analysis, a total of 110,279 effective sequences were obtained from each treatment. As shown in Table 1, CK, SF, and DSF treatments respectively got 10,104–12,502 (effective rate 92.90–96.28%), 10,779–12,530 (effective rate 92.16–96.40%), and 11,963–12,512 (effective rate 95.51–96.45%) after filtering the original data with Barcode tag sequence, optimized sequences (Table 1). The library coverage of samples processed by CK, SF, and DSF treatments were all high, which were 95.46%, 95.77%, and 96.34%, respectively. After quality control filtration, the number of sequences within the corresponding length range in each sample showed that almost all of the sequences ranged 1350–1650 bp in length and the average lengths of all samples ranged between 1452–1458 bp (Fig. 1). Our results showed that even if a deeper sequencing is carried out, there will be almost no more (operational taxonomic units) OTUs; that is, the sequencing library of the sample soil has reached a saturated state, and the constructed library contains most of the bacterial species in the sample, which can better reflect the soil bacterial community structure.

**Table 1:** Alpha diversity index of rice rhizosphere microorganisms under different fertilization modes of slow-release fertilizer.

Sample ID	Raw CCS	Clean CCS	Effective CCS	AvgLen (bp)	Effective (%)
CK1	13,073	12,502	12,145	1458	92.90
CK2	10,487	10,104	10,097	1454	96.28
CK3	11,515	11,047	11,026	1454	95.75
SF1	13,027	12,115	12,006	1454	92.16
SF2	12,990	12,530	12,522	1450	96.40
SF3	11,304	10,779	10,683	1455	94.51
DSF1	12,508	11,964	11,946	1452	95.51
DSF2	12,969	12,512	12,509	1457	96.45
DSF3	12,406	11,963	11,955	1456	96.36

*CCS* Circular Consensus Sequencing, Raw CCS was the number of CCS identified by the sample; Clean CCS was the number of sequences after primer removal and length filtering; Effective CCS was the number of sequences used for subsequent analysis after removal of chimera; AvgLen (bp) was the average sequence length of the sample; Effective (%) was the percentage of effective CCS in raw CCS.

### OTU analysis

**Figure 2:**
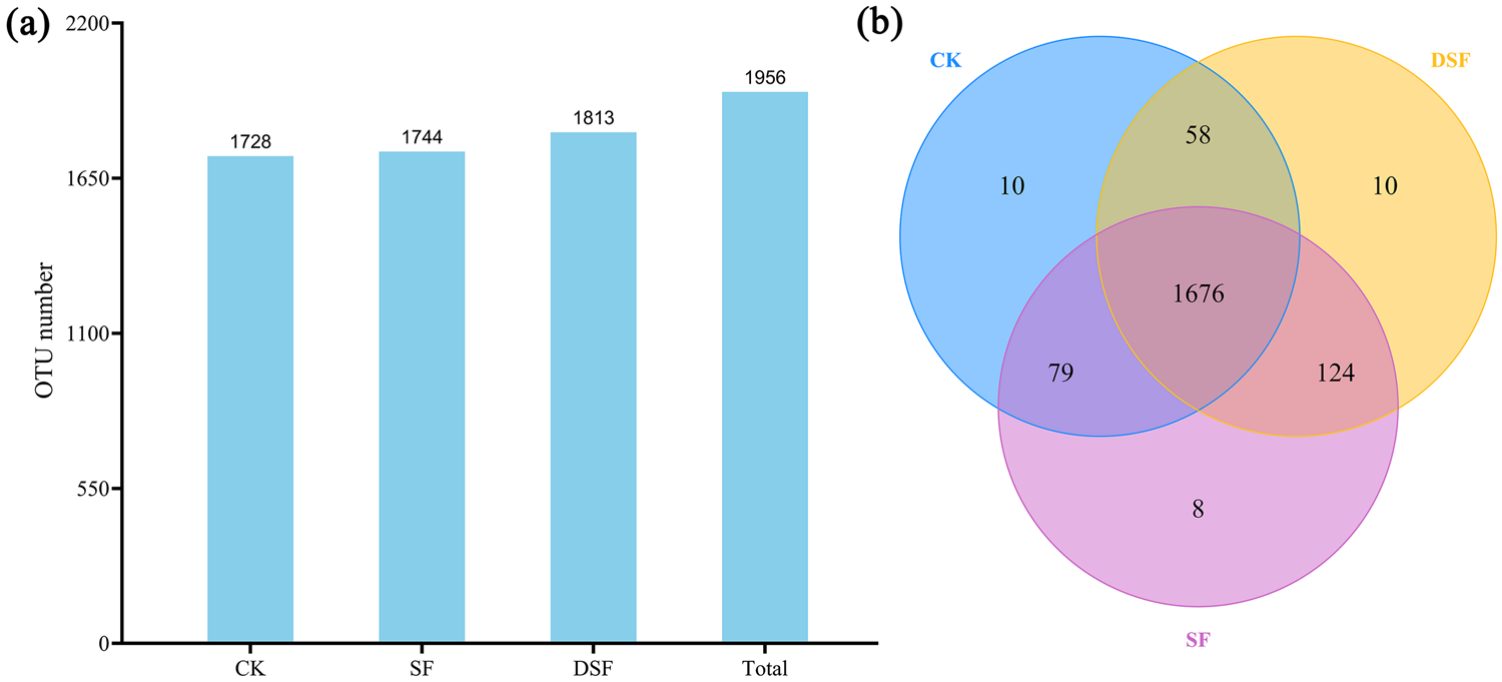
(**a**) The number of OTUs in different treatments. (**b**) The Venn map for the characteristic number of rice rhizosphere microorganisms under different fertilization modes of slow-release fertilizer. Values are the mean of three replicates.

OTU is the same marker set for a taxon (strain, species, genus, grouping, etc.) in phylogenetic or population genetics research to facilitate analysis. OTUs of all sequences can be divided according to different similarity levels, and each OTU corresponds to a representative sequence. As shown in Fig. 2a, there were 1956 OTUs in total, and the numbers of OTUs in CK, SF, and DSF treatments were 1728, 1744, and 1813, respectively. Figure 2b showed that there were differences in the characteristic number of rhizosphere microorganisms under different fertilization modes of slow-release fertilizer. The number of common characteristics among all samples was 1676. The number of unique features in CK, SF, and DSF treatments were 10, 8, and 10, respectively. There were 58 common characteristics only shared by CK and DSF treatments, and 79 common characteristics only shared by CK and SF treatments, while 124 common characteristics only shared by CK and DSF treatments only shared by SF and DSF treatments.

### Species number and distribution of rice rhizosphere microorganisms

**Figure 3:**
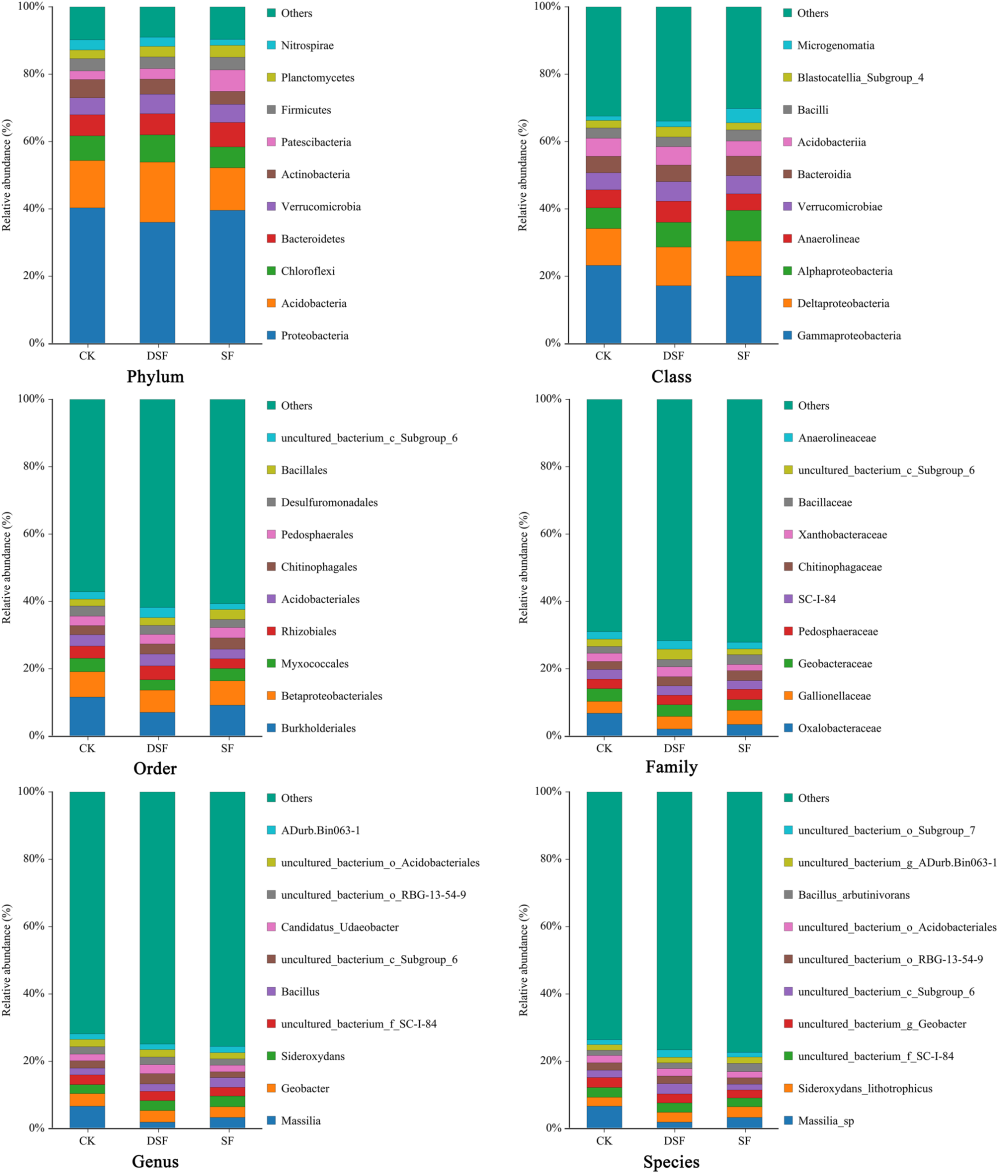
Species distribution at each level of rice rhizosphere microorganisms under different fertilization modes of slow-release fertilizer. Values are the mean of three replicates.

The number of species at each level of rice rhizosphere microorganisms under different fertilization modes of slow-release fertilizer were shown in Table 2, and Fig. 3 showed the species distribution at each level. At the level of phylum, the proportion of Acidobacteria in DSF treatment was higher than CK, and the proportion of Patescibacteria treatment in SF was much higher than CK. At the level of class, the proportions of Gammaproteobacteria in SF and DSF treatments were lower than CK, and the lowest proportion was recorded in DSF treatment. At the level of order, the proportions of others in SF and DSF treatments were higher than CK, while the proportions of Burkholderiales in SF and DSF treatments were lower than CK. At the level of family, the proportions of Oxalobacteraceae in SF and DSF treatments were lower than CK. At the level of genus, the proportions of *Massilia* in SF and DSF treatments were lower than CK. Moreover, at the level of species, the proportions of others in SF and DSF treatments were higher than CK, and the proportions of *Massilia_sp* in SF and DSF treatments were lower than CK while the lowest proportion was recorded in DSF treatment.

**Table 2:** The number of species at each level of rice rhizosphere microorganisms under different fertilization modes of slow-release fertilizer.

Treatment	Kindom	Phylum	Class	Order	Family	Genus	Species
CK	1	38.67	102.67	206.00	282.67	386.67	438.33
SF	1	38.67	103.67	204.67	282.33	393.00	444.00
DSF	1	36.67	102.67	204.33	285.33	392.00	446.00
Total	1	43	112	233	331	473	545

Values are the mean of three replicates.

### Effects of different fertilization modes of slow-release fertilizer on abundances of rice rhizosphere microorganisms at each level

**Figure 4:**
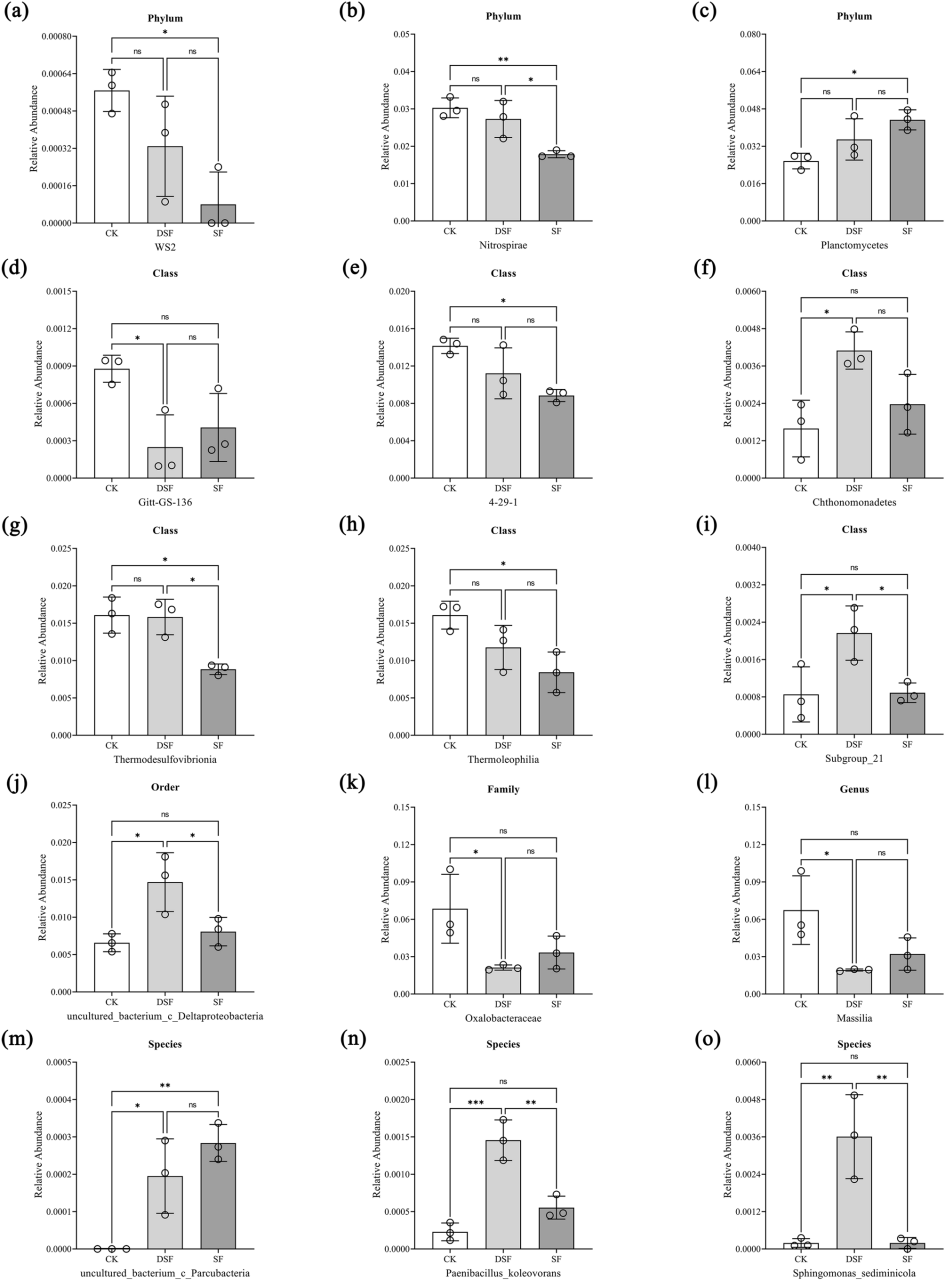
(**a–c**) The abundances of rice rhizosphere microorganisms at phylum level under different fertilization modes of slow-release fertilizer. (**d–i**) The abundances of rice rhizosphere microorganisms at class level under different fertilization modes of slow-release fertilizer. (**j–l**) The abundances of rice rhizosphere microorganisms at order, family, and genus level under different fertilization modes of slow-release fertilizer. (**m–o**) The abundances of rice rhizosphere microorganisms at species level under different fertilization modes of slow-release fertilizer. Group comparisons were done using one-way analysis of variance with Tukey’s multiple comparisons test. Calculated p-values less than 0.05 were determined to be statistically significant and indicated on graphs. Data are presented as mean ± SD. Statistical significance is considered as *p < 0.05, **p < 0.01, ***p < 0.001 and ns = p > 0.05.

As shown in Fig. 4, there were differences among different fertilization modes of slow-release fertilizer on abundances of rice rhizosphere microorganisms at each level. Compared with CK, SF treatment significantly increased the relative abundance of Planctomycetes and decreased the relative abundance of WS2 and Nitrospirae at the phylum level (Fig. 4a–c). Among them, Planctomycetes and Nitrospirae were the dominant phyla. Compared with CK, DSF treatment increased the relative abundance of Chthonomonadetes and Subgroup_21 significantly and decreased the relative abundance of Gitt-GS-136 at the class level significantly. Besides, lower relative abundances of 4-29-1, Thermoleophilia, and Thermodesulfovibrionia were recorded in SF and DSF treatments than CK, while the difference between CK and SF treatments reached a significant level (Fig. 4d–i). However, none of them was the dominant class. A significantly higher relative abundance of uncultured_bacterium_c_Deltaproteobacteria was recorded in DSF treatment than CK at the order level (Fig. 4j). Compared with CK, DSF treatment significantly decreased the relative abundance of Oxalobacteraccae at the family level and *Massilia* at the genus level (Fig. 4k,l). In addition, Oxalobacteraccae and *Massilia* were the dominant families and the dominant genera, respectively. Compared with CK, DSF treatment significantly increased the relative abundance of *Paenibacillus_koleovorans* and *Sphingomonas_sediminicola* at the species level (Fig. 4m). A significantly higher relative abundance of *uncultured_bacterium_c_Parcubacteria* was recorded in both DSF and SF treatments than CK (Fig. 4n,o).

### Alpha diversity analysis

As shown in Table 3, different fertilization modes of slow-release fertilizer influenced the indexes in Alpha diversity analysis. The numbers of feature in SF and DSF treatments were higher than CK, which were 1440.33, 1469.00, and 1365.00. Compared with CK, the values of ACE index increased in SF and DSF treatments by 2.50% and 3.45%. Compared with CK, SF and DSF treatments also increased the values of Chao1 index and Shannon index. In addition, 5.71% and 5.17% higher values of PD_whole_tree index were recorded in SF and DSF treatments than CK.

**Table 3:** Alpha diversity analysis of rice rhizosphere microorganisms under different fertilization modes of slow-release fertilizer.

Treatment	Feature	ACE	Chao1	Simpson	Shannon	PD_whole_tree	Coverage
CK	1365.00	1693.99	1663.52	0.9927	9.01	79.75	0.95
SF	1440.33	1736.40	1724.19	0.9937	9.15	84.31	0.96
DSF	1469.00	1752.54	1726.91	0.9962	9.24	83.88	0.96

Feature: the number of OTUs; ACE, Chao1, Simpson, Shannon, PD_whole_tree represent each index respectively; Coverage is the coverage rate of the sample library. Values are the mean of three replicates.

### Principal coordinates analysis (PCoA)

**Figure 5:**
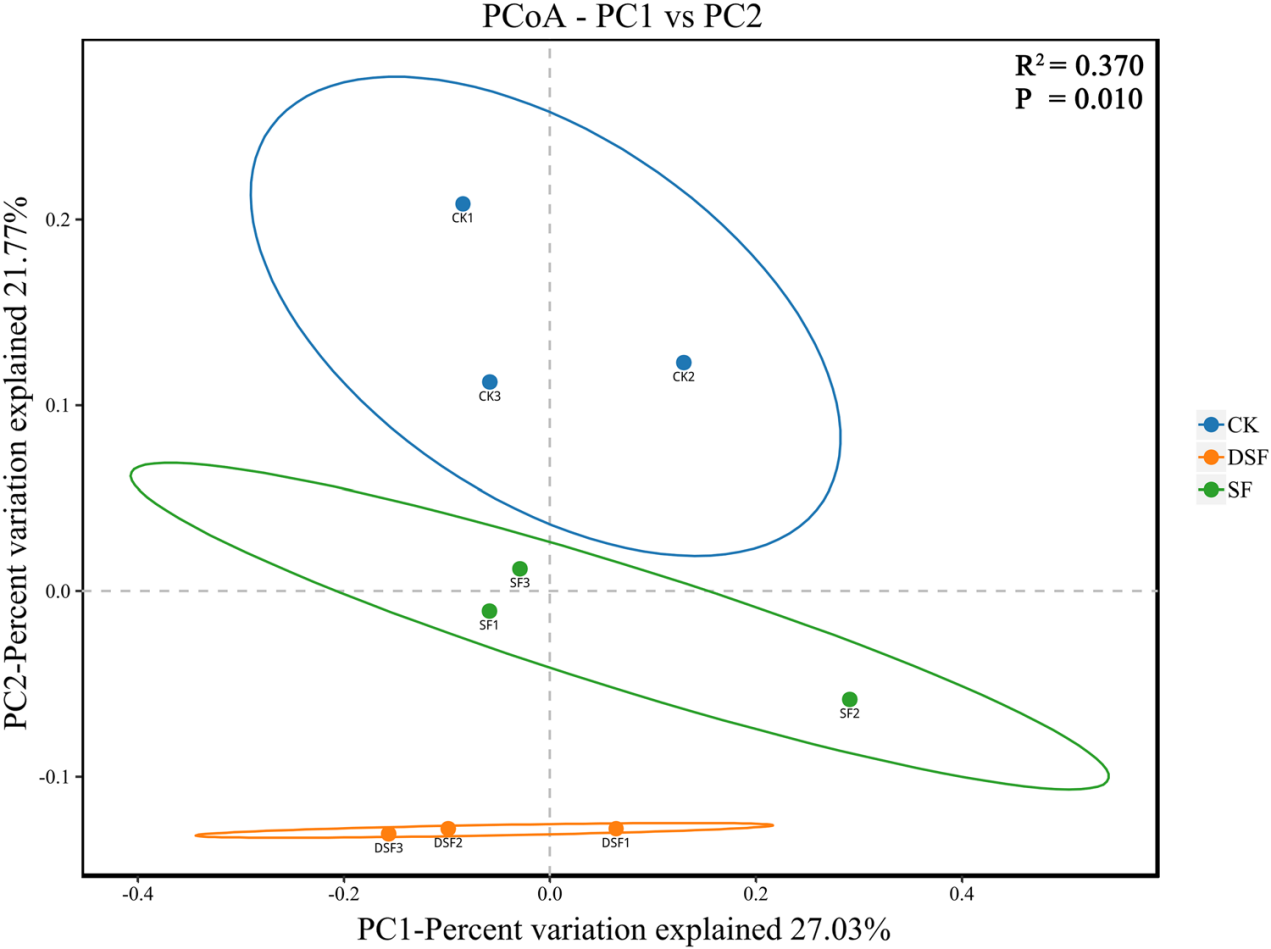
Bray–Curtis Principal coordinates analysis of rice rhizosphere microorganisms under different fertilization modes of slow-release fertilizer.

To explore whether the structural characteristics of microbial community composition in rice rhizosphere soil under different fertilization methods of slow-release fertilizer were dissimilar. Beta diversity was evaluated using Principal coordinates analysis (PCoA) based on the Bray–Curtis distance at the OTU level. The distribution of rhizosphere soil samples was discrete and did not gather together, the difference contribution rate of microbial community structure in the first principal component (PC1) sample was 27.03%, and the difference contribution rate of microbial community structure in the second principal component (PC2) sample was 21.77%. Figure 5 showed significant differences in bacterial communities among the samples (PCoA: PERMANOVA, P = 0.010 < 0.05).

### Partial factor productivity of applied fertilizer (PFP) and grain yield

The partial factor productivity of applied fertilizer (PFP) and grain yield were all different to the same extent under different fertilization modes of slow-release fertilizer (Table 4). The trend of grain yield was: SF > DSF > CK. The application amount of slow and controlled release fertilizer for both CK and SF treatments was 600 kg, but the SF treatment increased the yield by 35.92% compared with the CK treatment. The DSF treatment applied 20% less fertilizer than the CK treatment, but the DSF treatment increased the yield by 16.61% than the CK treatment. The trends of NPFP, PPFP and KPFP were all: DSF = SF > CK. Compared with CK treatment, the NPFP, PPFP, and KPFP of SF treatment all increased by 36.10%, the NPFP, PPFP, and KPFP of DSF treatment all increased by 45.83%.

**Table 4:** Partial factor productivity of applied fertilizer (PFP) and grain yield of different fertilization modes of slow-release fertilizer.

Treatment	NPFP (kg·kg^−1^)	PPFP (kg·kg^−1^)	KPFP (kg·kg^−1^)	Yield (t·ha^−1^)
CK	36.90 ± 1.05b	153.75 ± 4.38b	48.55 ± 1.38b	5.54 ± 0.15c
SF	50.22 ± 1.11a	209.25 ± 4.63a	66.08 ± 1.46a	7.53 ± 0.17a
DSF	53.81 ± 1.02a	224.22 ± 4.24a	70.81 ± 1.34a	6.46 ± 0.12b

NPFP is the partial factor productivity of applied nitrogen fertilizer, PPFP is the partial factor productivity of applied phosphate fertilizer, KPFP is the partial factor productivity of applied potash fertilizer; Mean ± SE sharing a common letter within a column don’t differ significantly at p < 0.05 according to Tukey’s multiple comparisons test.

## Discussion

Compared with normal chemical fertilizer, slow-release fertilizer with the regulated release of nutrients is an effective way to satisfy the demand for nutrients of crops during the whole growth stage^10^. The present study firstly revealed the microbial community characteristics in rice rhizosphere soil under different fertilization modes of slow-release fertilizer. Soil microbial diversity is rich, which is conducive to maintaining the stability of the soil ecosystem^29^. The diversity index is an important index to evaluate the diversity of the soil microbial community. The higher the diversity index is, the richer the diversity of the microbial community is, and the more uniform the distribution of microbial community is^30^. In our study, different fertilization modes of slow-release fertilizer affected the richness and diversity of rice rhizosphere bacteria. Compared with conventional broadcasting, deep placement of slow-release fertilizer increased the values of both ACE index and Chao1 index, indicating the richness of microorganisms was improved. Meanwhile, the higher values of Shannon index indicated that the deep placement of fertilizer increased the diversity of rice rhizosphere microorganisms. The study of Li et al.^31^. showed that fertilizer could indirectly affect the community structure of bacteria by affecting the utilization rate of fertilizer. Therefore, we speculated that the deep placement of slow-release fertilizers indirectly changed the bacterial community structure by improving the utilization rate of fertilizers.

The study of Wang et al.^32^ showed that the different fertilization modes had a significant influence on the community structure of bacteria but had no significant effect on the total levels of alpha diversity of soil bacteria. As shown in Table 5, there was no significant correlation between the different fertilization modes of slow-release fertilizer and their composition and α diversity index, which was consistent with the study of Wang et al.^32^. The number of OUTs and α diversity indices of the deep-placement slow-release fertilizer treatment were higher than those of the broadcast slow-release fertilizer treatment, but there were no significant differences (Table 3). However, a 22-year fertilization experiment^33^ demonstrated that the different fertilization modes significantly influenced the diversity of soil bacteria. The reason for the difference in the study results might be due to the length of time to apply different fertilization modes. Therefore, it is a long-term process to change soil microbial diversity through fertilization.

**Table 5:** Pearson correlation analysis of α diversity index, grain yield, PFP, and different fertilization modes and their composition.

Item	Deep placement	Amount	Modes
**α diversity indices**			
ACE indices	0.266	− 0.197	0.193
Chao1 indices	0.339	− 0.181	0.287
Simpson indices	0.333	− 0.445	0.127
Shannon indices	0.317	− 0.272	0.209
PD_whole_tree indices	0.397	− 0.169	0.361
**Grain yield and PFP**			
Grain yield	0.816**	0.043	0.967**
NPFP	0.959**	− 0.309	0.929**
PPFP	0.959**	− 0.309	0.929**
KPFP	0.959**	− 0.309	0.929**

“*” means significant correlation (p < 0.05), “**” means extremely significant correlation (p < 0.01); Deep placement indicates the deep application of slow-release fertilizer; Amount indicates the amount of slow-release fertilizer applied; Modes indicates the different fertilization modes of slow-release fertilizer.

The composition of soil microorganism community is affected by crops and soil environment while the composition and abundance of microorganism population in different farmland ecosystems are different^34^. The present study results showed that deep placement of fertilizer increased the relative abundance of Deltaproteobacteria, which was symbiotic and related to the utilization of many nutrients, such as soil organic matter, available phosphorus, and available potassium^35,36^. Higher relative abundance of Planctomycetes was also observed due to the deep placement of fertilizer. Bei et al.^37^ demonstrated that Planctomycetes could oxidize nitrite to ammonium ion and then produce nitrogen to obtain energy, which is of great significance to the global nitrogen cycle. Moreover, we noticed that deep placement of fertilizer reduced the relative abundance of Nitrospirae, which is the main microorganism in nitrosation reaction and can oxidize nitrite to nitrate^38^. Therefore, we deduced that the changes of relative abundances of Deltaproteobacteria, Planctomycetes, and Nitrospirae might be involved in regulating nitrogen cycling in rice rhizosphere soil under different fertilization modes of slow-releases fertilizer.

Interestingly, we observed that the deep placement of fertilizer increased the relative abundance of *Paenibacillus*. Chuang et al.^39^ demonstrated that *Paenibacillus* participated in the degradation of carbendazim in soil. The study of Luo et al.^40^ showed that *Paenibacillus* is one of the nitrate-reducing bacteria. The relative abundance of *Sphingomonas* also increased due to the deep placement of fertilizer. It might be because the deep placement of the fertilizer increased the surface area of the soil. The increase in the surface area of the soil allowed the oxygen to contact the soil more fully, which was conducive to the aerobic *Sphingomonas* to obtain more oxygen. The study of Premnath et al.^41^ showed that *Sphingomonas* could degrade high-molecular organic pollutants, which was conducive to environmental pollution control. Therefore, compared with broadcasting, that deep placement of fertilizer could not only reduce the loss of fertilizer and improve the utilization rate of fertilizer but also helped *Sphingomonas* degrade organic pollutants in farmland and further improve the quality of farmland.

Without harming the environment, high grain yield is the ultimate goal of rice production^42^. As shown in Table 5, both grain yield and PFP were extremely significantly positively correlated with the deep placement of slow-release fertilizer and the different fertilization modes of slow-release fertilizer. And there was no correlation between grain yield, PFP, and the amount of slow-release fertilizer applied, which indicated that the amount of fertilizer was not the key to increasing rice yield. Previous research has demonstrated that the number of biological nitrification inhibitors and nitrification inhibitors secreted by rice roots was positively correlated with rice ammonium absorption and preference^43^. As a result, deep placement of slow-release fertilizer had higher yields, which might be due to the nitrification inhibitors and the signaling compounds facilitating N-acquisition symbioses in root exudates that increase the fertilizer utilization^44–46^. Excessive application of nitrogen fertilizer would affect the composition and abundance of root exudates, thereby changing the community structure of soil bacteria, but it could not increase the abundance of beneficial microorganisms or reduce the abundance of undesirable microorganisms^47^. Compared with broadcasting, deep placement of slow-release fertilizer had a higher fertilizer utilization rate, increasing grain yield while reducing the amount of fertilizer used. Therefore, the deep placement of fertilizers is an environmentally friendly fertilization mode worth promoting in wetland rice planting.

## Conclusion

Different fertilization modes of slow-release fertilizer regulated the structure, distribution, and microbial community diversity in rice rhizosphere soil. Compared with broadcasting, deep placement of slow-release fertilizer improved the richness and diversity of rice rhizosphere microorganisms. Higher relative abundances of Deltaproteobacteria, Planctomycetes, and lower abundance of Nitrospirae were observed in the deep placement of fertilizer treatments. Deep placement slow-release fertilizer also increased relative abundances of *Paenibacillus* and *Sphingomonas*. Moreover, deep placement slow-release fertilizer had a higher fertilizer utilization rate and rice yield. Considering production and environmental factors, deep placement of 600 kg·ha^−1^ slow-release fertilizer (SF) is a higher yield fertilization mode, and deep placement of 480 kg·ha^−1^ slow-release fertilizer (DSF) is a fertilization mode with better environmental benefits.

## Materials and methods

### Plant materials and experimental site

*Meixiangzhan2*, selected and bred by the Rice Research Institute of Guangdong Academy of Agricultural Sciences, was widely cultivated for rice production in southern China^2^. It was planted on August 9, 2020, and harvested on November 17, 2020. At the maturity stage, the rice grains were harvested from a unit sampling area (50 m^2^) in each plot and threshed by machine. Then by weighing, the fresh weight yield of rice grains was obtained. The use of *Meixiangzhan2* in the present study complied with relevant institutional, national, and international guidelines and legislation.

This research was conducted at the Boluo county, Huizhou city, Guangdong Province, China (23° 52ʹ N, 114° 40ʹ E). The climate of the region was classified as subtropical monsoon climate, with hot, wet summers and warm, dry winters. The annual average temperature was 22.1 °C, the annual average rainfall was 1918.0 mm, the annual average sunshine was 1871.5 h, and the frost-free period was 357–362 d. The experimental soil in Boluo was Paddy soil in which pH was 6.3, containing 29.80 g·kg^−1^ organic matter, 1.61 g·kg^−1^ total N, 1.15 g·kg^−1^ total P, 3.84 g·kg^−1^ total K, 110.09 mg·kg^−1^ available N, 19.38 mg·kg^−1^ available P and 61.22 mg·kg^−1^ available K.

### Experimental details

A field experiment was conducted from August to November 2020. A special slow-release fertilizer for rice (N: P_2_O_5_: K_2_O = 25%: 6%: 19%) was applied in the present study. Three fertilizer treatments, i.e., (CK) manually broadcasted on the soil surface at 300 kg·ha^−1^ before transplanting and then same fertilizer rate was applied at the same way one week after transplanting; (SF) 10 cm depth mechanized placement at 600 kg·ha^−1^ during the transplanting; (DSF) 10 cm depth mechanized placement at 480 kg·ha^−1^ during the transplanting. Water management practices and pest management practices were followed as adopted by local farmers.

### Soil sample and pretreatment

Soil sampling was carried out when the rice was harvested. In each plot, five soil cores were randomly sampled for 0–20 cm soil with a 5.0 cm diameter of ring cutter soil drill. After removing the visible roots and crop residues, the soil cores were merged together to make composite samples on the same piece of land. Then, the mixed soil sample was immediately passed through a 2 mm sieve and stored at − 80 °C for DNA extraction.

### High-throughput sequencing

The structure of the soil microbial community was analyzed by 16SrDNA high-throughput sequencing method. TGuide S96 magnetic bead method soil genomic DNA extraction kit (Tiangen Biochemical Technology (Beijing) Co., Ltd.) was used to complete nucleic acid extraction. After extracting the total DNA of the sample, the 16S full-length forward primer 27F (5’-AGRGTTTGATYNTGGCTCAG-3’) and the 16S full-length reverse primer 1492R (5’-TASGGHTACCTTGTTASGACTT-3’) were used to synthesize specific primers with Barcode. Then the PCR amplification was performed, and the products were purified, quantified, and homogenized to form a sequencing library (SMRT Bell), which was sequenced with PacBio Sequel. Data preprocessing was carried out through lima v1.7.0, cutadapt 1.9.1, and UCHIME v4.2. The above operations were completed by Biomarker Technologies Corporation, Beijing, China.

### Partial factor productivity of applied fertilizer (PFP)

$${\text{NPFP }}\left( {{\text{partial factor productivity of applied nitrogen fertilizer}}} \right){\text{ }} = {\text{ Y}}_{{\text{N}}} /{\text{F}}_{{\text{N}}},$$

where Y_N_ is the grain yield (kg·ha^−1^) at a certain level of applied nitrogen fertilizer, and F_N_ is the rate of applied nitrogen fertilizer (kg·ha^−1^).

$${\text{PPFP }}({\text{partial factor productivity of applied phosphate fertilizer}}){\text{ }} = {\text{ Y}}_{{\text{P}}} /{\text{F}}_{{\text{P}}},$$

where Y_P_ is the grain yield (kg·ha^−1^) at a certain level of applied phosphate fertilizer, and F_P_ is the rate of applied phosphate fertilizer (kg·ha^−1^).

$${\text{KPFP }}({\text{partial factor productivity of applied potash fertilizer}}){\text{ }} = {\text{ Y}}_{{\text{K}}} /{\text{F}}_{{\text{K}}},$$

where Y_K_ is the grain yield (kg·ha^−1^) at a certain level of applied potash fertilizer, and F_K_ is the rate of applied potash fertilizer (kg·ha^−1^).

### Statistical analysis

The field experiment adopted a randomized complete block design, each treatment was repeated three times, and each plot area was 500 m^2^. Most high-throughput sequencing data were analyzed and graphed using the BMK Cloud (www.biocloud.net). The statistical comparisons of the rest of the data and corresponding p-values were calculated by Graphpad Prism 9 (Graph Pad Software, San Diego California USA, www.graphpad.com) through one-way analysis of variance with Tukey’s multiple comparisons test.
